# Preliminary Bioactivity Assessment of *Myrothecium* Species (Stachybotryaceae) Crude Extracts against *Aedes aegypti* (Diptera: Culicidae): A First Approach from This Phytopathogenic Fungi

**DOI:** 10.3390/jof10070466

**Published:** 2024-07-02

**Authors:** Carlos Granados-Echegoyen, José Abimael Campos-Ruiz, Rafael Pérez-Pacheco, Alfonso Vásquez-López, Ileana Vera-Reyes, Fabián Arroyo-Balán, Alberto Santillán-Fernández, Evert Villanueva-Sánchez, Clemente Villanueva-Verduzco, Alicia Fonseca-Muñoz, Fidel Diego-Nava, Yi Wang

**Affiliations:** 1 CONAHCYT-Instituto Politécnico Nacional, CIIDIR Unidad Oaxaca, Santa Cruz Xoxocotlán 71230, Mexico; 2 Facultad de Agronomía, Universidad para el Bienestar Benito Juárez García Oaxaca, Zaachila 71250, Mexico; josecampos92@gmail.com; 3 Instituto Politécnico Nacional, CIIDIR Unidad Oaxaca, Santa Cruz Xoxocotlán 71230, Mexico; rperezp@ipn.mx (R.P.-P.); avasquez@ipn.mx (A.V.-L.); 4 Biosciences and Agrotechnology Department, CONAHCYT-Center for Research in Applied Chemistry, Saltillo 25294, Mexico; ileana.vera@ciqa.edu.mx; 5 CONAHCYT-Centro de Estudios en Desarrollo Sustentable y Aprovechamiento de la Vida Silvestre (CEDESU), Universidad Autónoma de Campeche, San Francisco de Campeche 24079, Mexico; fabian.arroyo@conahcyt.mx; 6 CONAHCYT-Colegio de Postgraduados Campus Campeche, Champoton 24450, Mexico; santillan.alberto@colpos.mx; 7 Laboratorio Nacional de Investigación y Servicio Agroalimentario y Forestal, CONAHCYT-Universidad Autónoma Chapingo, Texcoco 56230, Mexico; evillanueva@conahcyt.mx; 8 Departamento de Fitotecnia, Universidad Autónoma Chapingo, Texcoco 56230, Mexico; clemente.villanueva@gmail.com; 9 Facultad de Sistemas Biológicos e Innovación Tecnológica, Universidad Autónoma Benito Juárez de Oaxaca, Oaxaca de Juárez 68120, Mexico; afonseca.cat@uabjo.mx; 10 Department of Analytical Chemistry, The Connecticut Agricultural Experiment Station, New Haven, CT 06504, USA; yi.wang@ct.gov

**Keywords:** phytopathogen, larvicide, fungi, mitospores

## Abstract

Mosquitoes, as insect vectors, play a crucial role in transmitting viruses and parasites, leading to millions of human deaths in tropical and subtropical regions worldwide. This study aimed to evaluate the effects of ethanolic extracts of three species within the genus *Myrothecium* (*M. roridum*, *M. dimerum*, and *M. nivale*) on *Aedes aegypti* mosquito larvae to assess the inhibitory effect on growth and development, as well as to determine mortality. We quantify the average lethal concentrations and provide a qualitative characterization of the chemical groups responsible for their potential. Phytochemical screening revealed the presence of alkaloids, flavonoids, and terpenoids in the ethanolic extracts of the three fungal species. Tannins were found only in the extracts of *M. dimerum* and *M. roridum*. We observed a clear dependence of the effects of the crude extracts on mosquito larvae on the concentrations used and the duration of exposure. The toxic effect was observed after 48 h at a concentration of 800 ppm for both *M. dimerum* and *M. nivale*, while *M. roridum* showed effectiveness after 72 h. All three species within the genus *Myrothecium* exhibited 100% biological activity after 72 h of exposure at 600 ppm. At lower concentrations, there was moderate growth and development inhibitory activity in the insect life cycle. The study highlights the effectiveness of crude *Myrothecium* extracts in combating mosquito larvae, with effects becoming apparent between 48 and 72 h of exposure. This initial approach underscores the potential of the fungus’s secondary metabolites for further in-depth analysis of their individual effects or synergies between them.

## 1. Introduction

Mosquitoes are insect vectors of viruses and parasites that cause millions of human deaths in various tropical and subtropical regions [1]. The transmission of arboviruses such as dengue and chikungunya is becoming an increasing public health concern in higher latitudes, including temperate regions [2]. Mosquito populations are expanding into new regions due to climate change. Due to rising global temperatures, the mosquito season is lengthening, allowing these disease vectors to expand into new regions [3].

A total of 41 genera and 3500 species have been described, of which 100 have been reported as vectors of disease in humans and other vertebrates. These Culicidae pose a significant public health concern as carriers of human diseases, such as malaria, filariasis, Japanese encephalitis, dengue, Zika, chikungunya, and yellow fever [4]. Their bites can trigger allergic reactions like urticaria or angioedema in humans [5].

The *Aedes aegypti* Linnaeus (Diptera: Culicidae) mosquito is the main vector responsible for transmitting arboviruses that cause several diseases, including dengue, yellow fever, and chikungunya [6]. Dengue fever has emerged as a significant health concern, with the number of reported cases rising; the more severe forms of the disease may result in hemorrhagic fever, dengue shock syndrome, and, sometimes, unusual manifestations involving the central nervous system. This disease is widespread in over 100 countries and poses a threat to the health of 2.5 billion people [7].

Dengue has seen a dramatic increase in global incidence over the past two decades, becoming a significant challenge. From 2000 to 2019, reported cases surged tenfold, from 500,000 to 5.2 million [8]. In 2023, the trend continued with an upsurge in cases characterized by significant increases and the simultaneous occurrence of multiple outbreaks, even in regions unaffected by dengue, like Northern Europe [9]. Some experts attribute the rise of dengue to factors such as increasing temperatures, extreme weather events, rapid population growth, and insufficient water and sanitation services. While there is no specific treatment for these diseases, early detection, appropriate case management, and preventive measures are crucial to reduce the burden of these arboviruses [10]. Public health efforts should continue to focus on surveillance, vector control, exploring active compounds in biodiversity, and raising community awareness to mitigate the impact of these diseases.

Strategies for controlling mosquito populations that transmit diseases often rely on the prolonged and indiscriminate use of synthetic chemical pesticides. However, this approach has negative impacts, including the development of resistance, toxicity to non-target organisms, destabilization of human health, and contamination of the environment [11]. Efforts are currently being made to identify resources that can be integrated into the management and control of these dipteran communities. This is aimed at reducing the mortality and morbidity they cause in humans and animals. Some of these alternatives include plant-derived products, such as essential oils and plant extracts, biological control agents like fish and nematodes, and secondary metabolites produced by other organisms, such as fungi and bacteria.

The phytopathogenic fungus *Myrothecium* Alb. & Schwein. (Hypocreales: Stachybotryaceae) comprises eight species, some of which exist as saprophytes in soil and are known to be pathogens of over 200 plant species [12]. Studies have demonstrated that fungi in this genus produce bioactive compounds, including terpenoids, diterpenes, amides, sterols, lactones, and myrothecosides, which have shown biological activity against different organisms [13,14,15,16]. There are precedents supporting the efficacy of this phytopathogenic fungus, which justify its study. As an example, the acetone extract of *M. verrucaria* was found to have nematicidal activity against Meloidogyne incognita (Heteroderidae) [17]. Additionally, the methanol extract derived from the solid mycelium of *Myrothecium* sp. SC0265 exhibited activity against Staphylococcus aureus, as reported by Fu et al. [15]. In addition, Ruma et al. [15] discovered that the crude ethyl acetate extract from the mycelium of *Myrothecium* sp. M1-CA-102 exhibited activity against *Shigella flexneri*, *Klebsiella pneumoniae* (Enterobacteriaceae), *Candida albicans* (Saccharomycetaceae), and plant pathogenic fungi such as *Rhizoctonia solani* (Agonomicetaceae) and *Fusarium oxysporum* (Nectriaceae) [18].

Recent studies have shown promise in using fungal secondary metabolites to control disease vectors, such as mosquitoes. Certain secondary metabolites produced by fungi have insecticidal properties [19]. These compounds can disrupt various physiological processes in mosquitoes, leading to their death or reduced ability to reproduce. Fungal compounds such as *Beauveria bassiana* [20], *Metarhizium anisopliae* [21], and *Isaria fumosorosea* [22] are effective against mosquito larvae. Fungal secondary metabolites can have varying effects on mosquitoes. The insect’s cuticle can be attacked by certain compounds, resulting in drying out and eventual death, while others disrupt vital processes such as development, reproduction, or immune function [23]. Utilizing fungal secondary metabolites to control disease-carrying mosquitoes is a promising and environmentally friendly approach that could support global efforts in combating mosquito-borne diseases.

This study aimed to evaluate the effects of ethanolic crude extracts of three Myrothecium species (*M. roridum*, *M. dimerum*, and *M. nivale*) on *Ae. aegypti* mosquito larvae. Additionally, we measured the mean lethal concentrations and analyzed the chemical groups contributing to their larvicidal properties.

## 2. Materials and Methods

### 2.1. Aedes aegypti Colonization

Mosquito eggs, larvae, and pupae were collected from natural and artificial reservoirs containing stagnant water in Campeche City, Mexico (19°49′36.7″ N, 90°33′15.6″ W). As part of the epidemiological surveillance conducted by the Health Department of this state, the immature stages were collected in December 2022. To minimize the development of resistance when introducing samples from the environment, the mosquitoes were reared for one year, undergoing successive complete cycles. The establishment of the bioassays was achieved using the F17 progeny, where susceptibility was compared with field samples by the Health Department. Samples were transferred to the Applied Entomology Laboratory of the Autonomous University of Campeche (UAC) and placed in plastic trays measuring 47 cm × 35 cm × 12 cm, with 300 mL of water inside a 40 cm × 40 cm × 40 cm entomological cage for the development of immature stages. Larvae were fed with ground tilapia fish food (Api-tilapia level 1^®^) until they reached the pupal stage. Pupae were transferred to containers measuring 30 × 20 × 20 cm and filled with 300 mL of water. The containers were then placed in entomological cages measuring 60 × 60 × 60 cm to allow for adult emergence. Adult males were fed a 10% sugar solution. After mating, an immobilized rabbit (*Oryctolagus cuniculus*, Leporidae) was introduced for one night as a source of blood for the female insects to promote egg-laying. Plastic containers with Whatman No. 1 filter paper attached to the walls were placed to collect mosquito eggs. The filter paper strips were collected and transferred to plastic containers with distilled water to encourage hatching and obtain larvae for bioassays. The larvae were maintained until the early second instar stage. The rearing was maintained in the laboratory at 27 ± 2 °C, 60–70% relative humidity, and a photoperiod of 12 h light and 12 h dark [24,25,26].

### 2.2. Obtaining Fungal Extracts Concentrations

Soriana^®^ brand rice was used as a culture medium to produce biomass of the phytopathogenic fungus. The rice was rinsed with distilled water, crushed with a mortar, and dried in a Felisa FE-291 oven at 50 °C. Twenty grams of the dried and crushed rice was then placed in 250 mL glass flasks with 30 mL of distilled water, and the mixture was allowed to ferment for 24 h. The material was then sterilized in a Yamato SM500 autoclave at 121 °C for 30 min.

Three fungal species (*M. roridum*, *M. dimerum*, and *M. nivale*) from the strain collection of the Center for Research in Environmental Microbiology and Biotechnology of UAC were used and cultured on Petri dishes with potato dextrose agar. After ten days of incubation at 28 °C, 10 mL of 0.85% NaCl saline with 0.1% Tween 80 (SST) was added to each dish. The surface of the Petri dish was then scraped to remove mycelium and spores. Then, 6 mL of the resulting mixture was decanted into 10-mL Falcon tubes to remove excess mycelium. From the filtered inoculum, 6 mL aliquots were taken, and the flasks were inoculated with rice and distilled water. Inoculated blanks were prepared using 6 mL of SST.

After 40 days of incubation at 28 °C, 100 g of rice with fungal growth was transferred to 500-mL Erlenmeyer flasks and macerated with 250 mL of 96% ethanol. The flasks were placed in a Lab-Line^®^ 4626 Orbiter with agitation at 120 revolutions per minute (rpm) for 24 h. The solution was then filtered through Whatman No. 1 filter paper, and the remaining solution was returned to the flask with the addition of solvent. This process was repeated twice. The filtrate was concentrated, and the solvent was removed using a Buchi B-490 rotary evaporator at 40 °C and reduced pressure for each species within the genus [27,28].

From the stock solution of the crude fungal extract, 880 mg was taken and dissolved in 11 mL of distilled water with 0.07% dimethyl sulfoxide (DMSO) as an emulsifier. The solution was used to prepare the extract application concentrations for each of the experimental units. Subsequent concentrations were prepared through serial volumetric dilution. The required amount of the initial concentration was mixed with distilled water containing 0.07% DMSO to achieve the desired application concentration in a total volume of 11 mL. This process was repeated until the lowest concentration was achieved. The test concentrations were 25, 50, 100, 200, 400, 600, and 800 ppm [29].

### 2.3. Phytochemical Analysis and Thin-Layer Chromatography (TLC)

Phytochemical Analysis and Thin-Layer Chromatography (TLC) were conducted to identify alkaloids, coumarins, anthraquinones, flavonoids, tannins, and terpenoids, following the methods outlined by Pascual et al. [30] and Mboneye et al. [31]. The presence of alkaloids was determined using Dragendorff’s reagent on the dry TLC plate, with the appearance of orange spots indicating their presence. Coumarins and anthraquinones were detected using a Bornträger solution (5% potassium hydroxide in EtOH). Coumarins were identified by the development of blue fluorescence at 365 nm, whereas anthraquinones were indicated by the presence of a red color. Flavonoids were identified through the application of diphenylboryloxyethylamine (NP) sprayed with 1% methanol, followed by polyethylene glycol (PEG) with 5% EtOH. The presence of flavonoids was confirmed by the observation of intense yellow, red, blue, or green fluorescence under 365 nm UV light. The presence of tannins was indicated by the appearance of dark, blue-black areas on TLC plates following the application of 3% ferric chloride. For the identification of terpenoids, the detection reagent developed by Godin was employed. This reagent consists of a 1% vanillin solution in ethanol, mixed in a 1:1 volume ratio with a 3% perchloric acid solution in water, followed by the application of a 10% sulfuric acid solution in ethanol. The plate was subjected to heating at a temperature of 105 °C for 3 min, and the presence of terpenoids was identified through the manifestation of a red or blue color. Then, 10 μL of the samples was deposited onto the chromatographic plates and subsequently subjected to development in a toluene/acetone (80:20) solvent system (*v*:*v*). The chromatograms were initially observed under 365 nm ultraviolet light without chemical treatment, followed by the application of sputtering reagents.

### 2.4. Larvicidal Bioassay

The effect of mortality induced by crude extracts on early second instar larvae of *Ae. aegypti* was recorded for three consecutive days. In addition, the development of the different stages (second, third, and fourth instars, as well as pupae and adults) was monitored for 20 days after the treatment application. During this period, the stages displaying mortality were quantified. These procedures were carried out according to the standardized guidelines of the World Health Organization [32]. Mortality was assessed based on two criteria: (1) absence of movements similar to those of the negative control group in a larva and (2) lack of response from a larva after being disturbed with a brush in the siphon of its cervical region.

### 2.5. Growth Inhibition

Early second instar larvae were selected for the bioassay due to their heightened sensitivity and susceptibility to experimental treatments. This enables a more accurate and dependable assessment of the inhibitory effects of the compounds under investigation. In addition, this specific instar was selected due to the uniformity in size and age of the larvae, which helps to minimize variability in the results. When the untreated control group produced between 90 and 93% pupae, we counted both viable and non-viable organisms (larvae and pupae), as well as the number of adults that emerged. An adult was considered dead if it remained trapped in the pupal exuvia. A diseased larva or pupa exhibited abnormal movements when disturbed with a dissection needle, following the methodology of Granados-Echegoyen et al. [33]. The information collected was used to quantify relative growth inhibition (RGI) according to the formula established by Zhang et al. [34].

$$RGI=\frac{\sum_{1}^{4}(No.\;of\;live\;insects*insect\;phase)+\sum_{1}^{4}[No.\;of\;dead\;insects*(insect\;phase-1)]}{\left(Total\;number\;of\;insects\;evaluated*total\;number\;of\;insect\;stages\right)}$$

where 1, 2, 3, and 4 correspond to the second, third, and fourth instar, and the pupal stages of the insect, respectively. The RGI data of mosquitoes treated with the fungal crude extract were grouped into categories, as follows: No inhibitory growth activity (RGI ≥ 1.00), Low inhibitory activity (0.75 ≤ RGI ≤ 0.99), Moderate inhibitory activity (0.50 ≤ RGI ≤ 0.74), High inhibitory growth activity (0.25 ≤ RGI ≤ 0.49), and Very high inhibitory growth activity (0.00 ≤ RGI ≤ 0.24).

### 2.6. Experimental Design and Statistical Analysis

The bioassays were conducted individually, following a completely randomized design for each variable under investigation. The experimental assays were set up by placing 20 larvae in plastic cups containing 99 mL of distilled water and 1 mL of crude fungal extract in triplicate for each of the 60 larvae tested. Each treatment was replicated three times, and two control groups were established. For comparison, untreated larvae were used as a negative control with an expected mortality rate of 0–4%, as recommended by Abbott in 1925. These controls were selected to assess the feasibility of the bioassay.

The normality of errors in the data was assessed using the Shapiro–Wilk test, and the homogeneity of variances was evaluated using the Bartlett test. One-way analysis of variance (ANOVA) was conducted, followed by a comparison of means using Tukey’s test at a significance level of *p* < 0.05 as a post hoc test. The statistical analysis was performed using Minitab version 20.3. Mortality data from each concentration test underwent Probit analysis to estimate LC_50_ and LC_90_ values [35]. The data presented display the means and standard deviations for the mortality and relative growth index variables. Lethal concentrations are expressed as means and standard errors of the mean, accompanied by 95% confidence intervals to account for a range of values that adhere to reliability standards.

## 3. Results

### 3.1. Phytochemical Screening and TLC Analysis

Phytochemical screening revealed that alkaloids, flavonoids, and terpenoids were present in the ethanolic extracts of the three fungal species, while tannins were found only in the extract of *M. dimerum* and *M. roridum* (Table 1).

### 3.2. Larvicidal Activity

A decrease in survival was observed with increasing concentrations of the extract and exposure times for all three crude extracts of the fungus. This suggests a dose- and time-dependent effect of *Myrothecium* extracts on the tested organism. For *Myrothecium dimerum*, a mortality rate of 13.33% was recorded after 24 h at the highest concentration (800 ppm), with a gradual increase in survival at lower concentrations. However, at 48 and 72 h, survival decreases to 100% at 800 ppm. The lethal concentrations (LC_50_ and LC_90_) for the first 24 h are 984.21 ppm and 1132.24 ppm, respectively. These concentrations improve significantly at 48 and 72 h, reaching values of 540.67 ppm and 611.07 ppm, and 419.59 ppm and 433.26 ppm, respectively. For *Myrothecium nivale*, larval mortality reached up to 83.33% within 24 h at the highest concentration (800 ppm), with a gradual decrease in mortality at lower concentrations. At 48 and 72 h, the survival rate decreased to 100% at 800 ppm. The median lethal concentrations at 72 h (LC_50_ and LC_90_) are 276.81 ppm and 394.01 ppm, respectively. *Myrothecium roridum* exhibits no survival at 800 ppm for 72 h, with lethal concentrations (LC_50_ and LC_90_) of 337.39 ppm and 565.77 ppm, respectively, for the first 48 h and LC_90_ of 209.31 ppm and 391.10 ppm, respectively, for 72 h (Table 2). The data analysis indicates that treatment with the *M. roridum* extract at a concentration of 200 ppm for 72 h is highly effective in reducing the survival of the organism, with an LC_50_ of 209.31 ppm and an LC_90_ of 391.10 ppm. This indicates that the treatment is highly effective in reducing the survival of the tested organism compared to the other treatments and concentrations evaluated.

### 3.3. Growth Inhibition

The relative growth index (RGI) indicates a reduction in the developmental time of the insect stages. The larvae of *Ae. aegypti* exposed to a concentration of 200 ppm of the ethanolic extract of *M. roridum* exhibited an RGI of 0.52, resulting in a 48% lower growth of the larval and pupal stages compared to the control (1.00). On the other hand, the growth inhibition effect was more pronounced in larvae exposed to concentrations of 400, 200, and 100 ppm of *M. dimerum* extract, with RGI values of 0.71, 0.77, and 0.80, respectively. The surviving insects spent less time in the pupal stage compared to the control group. The highest total mortality values were observed when larvae were exposed to the highest concentrations of each evaluated extract, while the lowest values were recorded with concentrations below 200 ppm for the extracts of *M. dimerum* and *M. nivale* (Table 3).

## 4. Discussion

Fungi of the genus Myrothecium have been found to have the ability to produce bioactive compounds from various chemical groups. Various studies have reported the presence of secondary metabolites, such as phenols, in the crude ethyl acetate extract obtained from *Myrothecium* sp. M1-CA-102 [15]. The presence of trichothecenes from extracts of *M. verrucaria* [36] and *M. roridum* [37], as well as sesquiterpenes from *Myrothecium* sp. SC0265 [15], which belongs to the terpenoid group and was identified in our study, was also demonstrated.

The larvicidal activity of metabolite filtrate from other fungal species, such as *Lecanicillium* (=*Verticillium*) *lecanii* (Cordycipitaceae), against *Culex quinquefasciatus* (Culicidae) has been evaluated, and the results show that fourth instar larvae are more susceptible than other instars after 72 h of exposure to treatments [38]. Similar results were obtained in our study with concentrations of 800 and 600 parts per million (ppm). Al-Mekhlafi [39] assessed the methanolic extract of *Paecilomyces lilacinus* (Ophiocordycipitaceae), which demonstrated 100% mortality in fourth instar larvae of *Ae. caspius* (LC50 of 190.66 ppm) and *Cx. pipiens* showed an LC50 of 254.25 ppm after 24 h of exposure to treatments, which is lower than the values obtained in our study. On the other hand, Ragavendran and Natarajan [40] assessed the ethyl acetate extract of *Aspergillus terreus* (Trichocomaceae). They found that at a concentration of 500 ppm, it caused mortality in second instar larvae and pupae of *Ae. aegypti* and *Culex* spp. Larvicidal activity of *Cx*. *quinquefasciatus* began within the first 6 h of exposure and exceeded 90% after 24 h. These findings differ from those observed with ethanolic extracts of *Myrothecium*, as the highest larvicidal activity was achieved with *M. nivale*, while the greatest pupal mortality occurred with a concentration of 200 ppm of the same extract.

Mitospores fungi, such as *Myrothecium*, can produce a wide variety of secondary metabolites synthesized by specialized metabolic pathways within the fungal cells [19]. The production of these metabolites is primarily determined by the fungal genome [41]. The genes responsible for synthesizing them encode enzymes that catalyze various chemical reactions, including polyphenol oxidase, dehydrogenases, and methyltransferases [42]. These genes are regulated through mechanisms such as activation or repression of messenger RNA transcription, post-transcriptional regulation, epigenetic regulation, and enzyme regulation, among others, which can modify their production [43].

Furthermore, environmental factors such as nutrient availability, temperature, humidity, and the presence of other organisms can influence the production of secondary metabolites in fungi [44]. Some secondary metabolites may be produced in response to stressful conditions, such as competition with other microorganisms or oxidative stress. Interactions with other organisms, such as plants, bacteria, or other fungi, may also influence their production [45].

During the phytochemical screening, alkaloids were detected in all three ethanolic extracts evaluated, which could potentially affect the observed efficacy. These compounds act as neurotoxins in insects, disrupting the normal functioning of their nervous system [46]. For example, they can inhibit cholinesterase, an enzyme essential for the transmission of nerve signals, leading to neuronal hyperactivity and ultimately causing paralysis and death in the insect. Some alkaloids can also disrupt insect metabolic processes, such as cellular respiration or protein synthesis, leading to death or interfering with normal development, including molting, metamorphosis, or reproduction [47].

Additionally, flavonoids were identified in the three crude extracts. These compounds possess insecticidal properties that can have a detrimental effect on insects [48]. These compounds can disrupt crucial physiological processes in insects, such as cellular respiration and larval development [49]. Some flavonoids can also impact the digestion of insects by inhibiting the activity of specific digestive enzymes, thereby reducing their ability to obtain nutrients essential for their development and weakening their overall health [50].

Terpenes/terpenoids have also been identified in the extracts of the three species of phytopathogenic fungi, which can disrupt the functioning of insects’ physiological systems and impact their normal growth and development [51]. Some terpenoids can act as hormone mimics or inhibitors of key enzymes in hormone synthesis, resulting in physiological dysfunction in insects [52].

Tannins were found in two out of the three fungal species studied. Myrothecium nivale was the only fungal extract that did not contain tannins but had the highest degree of efficacy in the shortest time. This suggests that the presence of tannins in extracts containing alkaloids, flavonoids, and terpenes may accelerate their efficacy. This could be due to the synergistic effects of secondary compounds in the extract, which may enhance or diminish the effectiveness of the mixture compared to the individual effects of each compound [53]. However, further research must complement the identification of the specific compounds responsible for the biological activity. The assessment of the synergistic effects of phytochemicals in an extract is intricate and necessitates a comprehensive analysis of their chemical composition and biological impacts. Synergistic effects may be unique to each combination of compounds and can vary depending on the extraction conditions and the concentrations of the components present [54].

There is no evidence of the effect of *Myrothecium* fungal extracts on mosquito larvae. However, there is evidence for the use of secondary metabolites from plants containing the chemical groups identified in our study. For example, there are reports on *Cx. quinquefasciatus* mosquito larvae of the species which were exposed to a methanol extract of Ricinus communis (Euphorbiaceae) seeds, resulting in a shortened larval period at a concentration of 162.5 ppm [55]. On the other hand, Daniel et al. [56] reported that extracts of *Acalypha indica* (Euphorbiaceae) increased the larval period, which is consistent with the data obtained in this study. The RGI was not evaluated using fungal extract concentrations. However, this index has been determined using botanical extracts, as reported by Granados-Echegoyen et al. [33]. They evaluated the application of 10% ethanol and methanol extracts of *Pseudocalymma alliaceum* (Bignonaceae) leaves on second instar larvae of *Cx. quinquefasciatus.* The study involved and reported RGI values of 0.70 and 0.76, respectively, which are similar to those obtained in this study with the application of concentrations ranging from 200 to 50 ppm of *M. nivale* crude extract.

The fact that different species of plants and fungi can contain the same chemical groups in varying amounts is a key factor that contributes to their effectiveness for specific biological functions and is closely related to the concept of chemical biodiversity [57]. This principle underscores the importance of researching the discovery of compounds present in these organisms, as it suggests that a diverse range of fungi, plants, and other organisms may contain compounds with valuable properties. This implies that exploring different species presents an opportunity to discover new compounds with potential applications. While different species may contain the same chemical groups, the quantity and concentration of these compounds may vary. Studying a variety of organisms enables the identification of sources with higher concentrations of compounds of interest, which can be valuable for optimizing the extraction and production of these compounds.

The presence of identical chemical groups in different species may indicate that these compounds play crucial roles in their biology. This is evident in the case of fungi and plants, where these compounds serve functions such as defense against predators, competition with other species, and adaptation to the environment. This provides valuable insights into how species have evolved to survive and adapt to diverse environmental conditions [58,59].

Vivekanandhan et al. [60] observed that fungal extracts have a significant effect on mosquito larvae, inducing vacuolization in the epithelium as well as in the fat and muscle tissues of the larval midgut. This effect makes them effective tools for controlling the larval stages of mosquitoes, such as *An. stephensi*, *Ae. aegypti*, and *Cx. quinquefasciatus*. Additionally, these extracts impact the gut microbiota of the larvae, altering their balance and indirectly influencing their health and development.

Fungal secondary metabolites have toxic properties that can interfere with vital physiological processes in mosquito larvae, such as digestion, respiration, and development, leading to toxicity and ultimately death. These metabolites can also act as endocrine disruptors, interfering with the hormonal system of mosquito larvae and affecting their growth, reproduction, or normal development [61].

Baskar et al. [62] documented significant histopathological changes in *Ae. aegypti* and *Cx. quinquefasciatus* exposed to *Aspergillus tamarii* mycelial extracts. They observed damaged nerve cord ganglia, disorganized gastric caeca, muscles, and alimentary spine in *Ae. aegypti*, and lesions with edema, swelling, and deformation of epithelial cells in *Cx. quinquefasciatus.* They also reported hyperplasia of epithelial cells in some regions of the tissues, contrasting the normal development observed in control larvae of both mosquitoes. These observations support the larvicidal effect found in our study.

The same authors have also observed a decrease in acetylcholinesterase (AChE) activity in *Cx. quinquefasciatus* and *Ae. aegypti* larvae, leading to an accumulation of acetylcholine at the synapse. This phenomenon results in overstimulation of cholinergic receptors and eventual muscle paralysis, ultimately causing the death of the insect [63]. Additionally, a decrease in the activity of carboxylesterase, an enzyme crucial for the metabolism and detoxification of xenobiotic compounds, has been noted, which may be associated with insecticide resistance.

It is important to note that the efficacy and specific effects of fungal secondary metabolites can vary considerably depending on the fungal species, the mosquito species, the environmental conditions, and the concentration of the metabolite present. Chemical compounds found in nature have a wide range of potential applications in medicine, agriculture, industry, and other fields. Understanding chemical biodiversity and discovering new compounds opens up opportunities for developing new natural pesticides and innovative materials.

## 5. Conclusions

Our study yielded promising results, demonstrating that ethanolic extracts derived from *M. dimerum*, *M. roridum*, and *M. nivale* possess significant toxic activity against *Ae. aegypti* mosquito larvae. Even at lower concentrations, a significant prolongation of the insect life cycle was observed, suggesting that the decrease in mosquito population could be substantial despite minimal larvicidal potential. Our preliminary phytochemical analysis revealed the major chemical groups present in the extracts and their biological activity, representing an initial step towards utilizing the potential of secondary metabolites from these phytopathogenic fungi in the integrated management of mosquito-borne diseases. It is essential to conduct additional research to compare the effectiveness of natural formulations with synthetic ones. Future studies should include evaluating solvent-based extracts with different polarities, as well as purifying, identifying, and characterizing the active constituents responsible for controlling mosquito larvae and inhibiting their growth.

## Figures and Tables

**Table 1:** Phytochemical constituents of ethanolic extracts of *Myrothecium* spp.

Metabolites	Reactive	*M. dimerum*	*M. nivale*	*M. roridum*
Alkaloids	Dragendorff	+	+	+
Coumarins	Bornträger	-	-	-
Anthraquinones	Bornträger	-	-	-
Flavonoids	NP/PEG ^1^	+	+	+
Tannins	FeCl3	+	-	+
Terpenoids	Godin	+	+	+

^1^ NP: Natural products; PEG: Polyethyleneglycol.

**Table 2:** Regression analysis and quantified lethal concentrations for three consecutive days of the effect of crude extracts of *Myrothecium* sp. on the larvae of the mosquito *Aedes aegypti* (Diptera: Culicidae).

Fungi Crude Extract	Days (h)	LC_50_ (CI)	LC_90_ (CI)	Slope (SE)	x^2^ (df)	z-Value	*p*-Value
*M. dimerum*	24	984.21 ± 8.21 (876.70–1362.78)	1132.24 ± 124.45 (975.66–1727.85)	−8.34 (1.88)	1.36 (5)	−4.44	<0.001
	48	540.67 ± 10.79 (514.71–559.44)	611.07 ± 10.05 (593.00–634.50)	−9.58 (1.45)	0.19 (5)	−6.59	<0.001
	72	419.59 ± 2660.09 (*)	433.26 ± 4516.23 (*)	−37.20 (4767)	0.00002 (5)	−0.01	0.994
*M. nivale*	24	446.85 ± 19.08 (410.30–485.99)	733.90 ± 32.00 (677.74–805.55)	−1.99 (0.15)	34.76 (5)	−12.84	<0.001
	48	325.22 ± 13.85 (298.86–354.00)	481.44 ± 21.69 (443.75–530.82)	−2.66 (0.23)	8.60 (5)	−11.58	<0.001
	72	276.81 ± 11.96 (254.18–302.02)	394.01 ± 18.59 (362.08–437.15)	−3.02 (0.28)	5.36 (5)	−10.67	<0.001
*M. roridum*	24	1007.41 ± 101.12 (888.49–1741.03)	1247.90 ± 189.28 (1032.26–2665.45)	−5.36 (1.53)	0.04 (5)	−3.50	<0.001
	48	337.39 ± 16.30 (306.50–371.25)	565.77 ± 27.42 (517.84–627.47)	−1.89 (0.14)	5.83 (5)	−12.79	<0.001
	72	209.31 ± 13.15 (184.82–237.43)	391.10 ± 25.25 (348.39–450.51)	−1.47 (0.13)	6.71 (5)	−10.77	<0.001

LC: lethal concentration; LC_50_: lethal concentration that controls half the pest population; LC_90_: lethal concentration that controls 90% of the population. CI: confidence intervals. SE: standard error; x^2^ (df): chi-squared value (degrees of freedom); z-value: test statistic value (z score); *p*-value: probability value.; (*): No LC determination. Does not reach convergence for the calculation criterion. A low number of iterations.

**Table 3:** Total mortality and relative growth index of *Aedes aegypti* mosquito larvae treated with ethanol extracts of *Myrothecium* spp.

Concentration (ppm)/Fungus	*M. dimerum*		*M. nivale*		*M. roridum*	
	TM	RGI	TM	RGI	TM	RGI
800	100.00 ± 0.00 a	-	100.00 ± 0.00 a	-	100.00 ± 0.00 a	-
600	100.00 ± 0.00 a	-	100.00 ± 0.00 a	-	100.00 ± 0.00 a	-
400	11.67 ± 2.89 b	0.71 ± 0.02 e	100.00 ± 0.00 a	-	100.00 ± 0.00 a	-
200	5.00 ± 0.00 c	0.77 ± 0.01 d	45.00 ± 5.00 b	0.68 ± 0.01 c	58.33 ± 7.64 b	0.52 ± 0.04 d
100	0.00 ± 0.00 d	0.80 ± 0.01 cd	0.00 ± 0.00 c	0.79 ± 0.01 b	36.67 ± 10.41 c	0.63 ± 0.07 c
50	0.00 ± 0.00 d	0.84 ± 0.01 bc	0.00 ± 0.00 c	0.79 ± 0.01 b	13.33 ± 2.89 d	0.82 ± 0.02 b
25	0.00 ± 0.00 d	0.86 ± 0.01 b	0.00 ± 0.00 c	0.80 ± 0.01 b	5.00 ± 0.00 de	0.90 ± 0.02 ab
Control (-)	0.00 ± 0.00 d	1.00 ± 0.02 a	0.00 ± 0.00 c	1.00 ± 0.02 a	0.00 ± 0.00 e	1.00 ± 0.02 a

Data represent means of three replicates (*n* = 3). Means followed by different letters (a, b, c, d, e) within the same column and fungal ethanolic crude extract are significantly different at *p* < 0.05. RGI: relative growth index. No inhibitory growth activity (RGI ≥ 1.00), Low inhibitory activity (0.75 ≤ RGI ≤ 0.99), Moderate inhibitory activity (0.50 ≤ RGI ≤ 0.74), High inhibitory growth activity (0.25 ≤ RGI ≤ 0.49), and Very high inhibitory growth activity (0.00 ≤ RGI ≤ 0.24).

## Data Availability

All the associated data are available in the manuscript.
